# A cuproptosis-related signature predicts prognosis and indicates cross-talk with immunocyte in ovarian cancer

**DOI:** 10.1007/s12672-024-00981-7

**Published:** 2024-05-02

**Authors:** Bikang Yang, Juan Yang, Keqiang Zhang

**Affiliations:** Department of Gynecologic Oncology, Hunan Cancer Hospital, The Affiliated Cancer Hospital of Xiangya School of Medicine, Central South University, 283 Tongzipo Road, Yuelu District, Changsha, 410013 Hunan People’s Republic of China

**Keywords:** Cuproptosis, Immunocytes infiltration, Chemotherapy, Prognostic signature, Ovarian cancer

## Abstract

**Purpose:**

Cuproptosis, programmed cell death by intracellular copper-mediated lipoylated protein aggregation, is involved in various tumorigenesis and drug resistance abilities by mediating the tumor microenvironment. Previous studies have demonstrated that serum copper levels are higher in OC patients than in normal subjects. However, the exact relationship between cuproptosis and ovarian cancer progression remains to be further elucidated.

**Methods:**

The Cancer Genome Atlas (TCGA) and gene expression omnibus (GEO) datasets were utilized to establish a cuproptosis-related prognostic signature in ovarian cancer. Subsequently, the bulk RNA-seq analysis and single-cell RNA-seq analysis were used to identify the relationship between signature with immune cell infiltration, chemotherapy, and cuproptosis-related scoring (CuRS) system. Finally, the potential biological functional roles of target genes in cuproptosis were validated in vitro.

**Results:**

By using LASSO-Cox regression analysis to establish the cuproptosis-related prognostic model, our works demonstrated the accuracy and efficiency of our model in the TCGA (583 OC patients) and GEO (260 OC patients) OC cohorts, and the high-scoring groups showed worse survival outcomes. Notably, there were substantial differences between the high and low-risk groups in extensive respects, such as the activating transcription factors, cell pseudotime features, cell intercommunication patterns, immunocytes infiltration, chemotherapy response, and potential drug resistance. KIF26B was selected to construct a prognostic model from the identified 33 prognosis-related genes, and high expression of KIF26B predicted poorer prognosis in ovarian cancer. Ultimately, further in vitro experiments demonstrated that KIF26B participated in the proliferation and cisplatin resistance of OC cells. Knockdown of KIF26B increased the sensitivity of OC cells to elesclomol, a cuproptosis agonists.

**Conclusion:**

This study constructed a new cuproptosis-related gene signature that has a good prognostic capacity in assessing the outcome of OC patients. This study enhances our understanding of cuproptosis associated with ovarian cancer aggressiveness, cross-talk with immunocytes, and serves as a novel chemotherapy strategy.

**Supplementary Information:**

The online version contains supplementary material available at 10.1007/s12672-024-00981-7.

## Introduction

Ovarian cancer (OC) is one of the most common malignant gynecological tumors, and its mortality rate remains high [1]. Because the disease is often asymptomatic and there is a lack of related biomarkers, a large proportion of patients are diagnosed at an advanced stage [2]. Despite the application of standard therapy such as debulking surgery and adjuvant chemotherapy, more than 70% of patients exhibit tumor recurrence due to chemotherapy resistance [3]. There has been tremendous research effort, and numerous treatment options, such as poly ADP-ribose polymerase (PARP) inhibitors and immunotherapy for ovarian cancer patients, have been tested [4, 5]. However, the molecular heterogeneity at the genomic level, prominent toxicity, and possible acquisition of drug resistance substantially decrease efficacy in the clinical applications of adjuvant chemotherapy and targeted therapies [6]. Therefore, in addition to the basic strategy of complete tumor resection, efficient therapeutic targets for sensitization and signatures of ovarian cancer related to the response to targeted therapy and immunotherapy should be explored to improve prognosis and survival.

Copper is an essential trace metal for living organisms (bacteria, animals, and humans) and plays an important role as a cofactor of copper-binding enzymes in many biological processes, including mitochondrial respiration, iron uptake, antioxidant activity, and detoxification [7]. Copper maintains dynamic equilibrium at low concentrations via evolutionarily conserved homeostatic mechanisms and participates in cell proliferation (cuproplasia), while abnormal accumulation of copper can cause cytotoxicity and induce cell death (cuproptosis) [8]. Copper ions induce abnormal oligomerization of lipoylated proteins by binding lipoylated components of the tricarboxylic acid (TCA) cycle, subsequently reducing iron-sulfur cluster protein levels and inducing a proteotoxic stress response, ultimately leading to cell death [9]. Significantly elevated copper levels in serum and tumor tissue have been found in patients with a variety of cancers, including breast cancer, thyroid cancer, pancreatic cancer, and bladder cancer [10–13]. An increasing number of studies have demonstrated that an abnormal increase in copper stress is involved in the proliferation, invasion, and metastasis of cancers [14]. Léo Aubert found that the copper-exporter ATP7A is essential for neoplastic growth by regulating intracellular copper icon levels and further indicated that copper bioavailability is a KRAS-specific vulnerability in colorectal cancer[15]. Additionally, copper ions contributed to tumor angiogenesis by activating many angiogenic factors, such as vascular endothelial growth factor (VEGF), VEGF2, and fibroblast growth factor 1 (FGF1) [16–18]. However, some studies showed that the prognosis of OC patients was correlated to the procuproptosis gene-lipoic acid synthase (LIAS), and patients with OC who have high expression of LIAS showed a better overall survival and post-progressive survival [19]. This demonstrated that promoting cuproptosis was beneficial to the prognosis of OC, but whether more CRGs had a positive effect on the prognosis of OC needed to be further explored.

In the present study, a scoring model based on cuproptosis-related differentially expressed genes was constructed, and this model may provide novel potential strategies to predict prognosis, immune infiltration, immunotherapy, and chemotherapy response. Furthermore, ligand–receptor pairs and RNA velocity in single-cell RNA-seq analysis were assessed based on this system, and these strategies supplement the current methods for evaluating novel connections between cancer cells and immunocytes. In general, the cuproptosis-related scoring model might assess aggressiveness and provide more efficient immunotherapeutic strategies for OC patients.

## Materials and methods

### Data preparation

The copy number variation (CNV), clinical information, and mRNA sequence data of ovarian cancer are downloaded from The Cancer Genome Atlas (TCGA) database (https://portal.gdc.cancer.gov/). This samples from the Gene Expression Omnibus (GEO) (https://www.ncbi.nlm.nih.gov/geo/) database are set as validation cohort, including clinical information and mRNA sequence. There are 583 OC patients in the training cohort from the TCGA dataset and 260 OC patients from the GEO dataset (GSE32062).

Data for single-cell RNA-seq (scRNA-seq) analysis is downloaded from the GEO database (GSE173682 and GSE158937) (https://www.ncbi.nlm.nih.gov/geo/query/acc.cgi). Expression data are normalized with R packages ‘Seurat’ and ‘NormalizeData’. The distribution of cells components is mapped with R package ‘UMAP’ [20].

### Unsupervised clustering for cuproptosis-related genes

Initially, the ten cuproptosis-related genes (CRGs) were identified from previous studies (PMID:35298263). Basis on the expression profiles of these 10 CRGs, 583 OC patients from the TCGA cohort were identified two clusters using the unsupervised clustering analysis, which used consensus clustering algorithm, and a follow-up analysis was conducted. TCGA-OV counts data are converted into transcripts per kilobase million (TPM) by using the R package “GeoTcgaData”.

### Functional enrichment analysis and immune cell infiltration analysis

The Gene Ontology (GO), Kyoto Encyclopedia of Genes and Genomes (KEGG) pathway, and Gene set variation analysis (GSVA) were utilized for enrichment analysis for bulk RNA-seq and scRNA-seq, to investigate the differences in biological processes and pathways among the differences cuproptosis-related scoring (CuRS) clusters [21]. The hallmark gene (c2.cp. Kegg.v7.2) was derived from the MSigDB database to run GSVA enrichment analysis. The single sample gene set enrichment analysis (ssGSEA) algorithm of the R package “GSEAbase” was performed to elucidate the enrichment of two different risk subgroups in 29 gene sets related to immune function [22]. CIBERSORT, a versatile computational method for quantifying cell fractions from bulk tissue gene expression profiles (GEPs), and can accurately estimate the immune composition of a tumor biopsy [23]. Subsequently, the Estimate algorithm is used to describe the infiltration rate of immune cells and stromal cells [24]. *p* < 0.05 were considered to indicate significant differences.

### Construction of a prognosis signature associated with cuproptosis

The deferentially expressed genes (DEGs) between the cuproptosis clusters are identified using the DESeq2 R package (*p* < 0.05 and |log2FoldChange|> 0.58). Primarily, DEGs are subjected to univariate Cox regression analysis to identify these 33 prognostic-related DEGs. Afterward, utilizing the “glmnet” R package, the LASSO Cox regression algorithm is recommended to minimize the risk of over-fitting depended on CRG prognostic genes [25]. In the TCGA training group, the multivariate Cox analysis identify 21 candidate genes and their correlative coefficients acquired for constructing the prognostic risk score. The following formula was used to determine the risk score:

$${\text{GuAscore = }}\sum\limits_{i = 1}^{n} {\exp ression\,of\,gene\,i\,*\,lasoo\,coefficient\,of\,gene\,i}$$

The patients in TCGA and GSE32062 OC cohort are further stratified as high-risk or low-risk subgroups, and then Kaplan–Meier survival analysis is performed, and the development of receiver operating characteristic (ROC) curves using the survival R package “timeROC” used to assess the predictive power of the signature [26]. Furthermore, applying the univariate and multivariate analyses on the clinical variables in training and GSE32062 groups to assess whether CRRS could be served as an independent prognostic factor.

### Evaluation of the sensitivity of chemotherapy and targeted therapy drugs

We downloaded from Genomics of Drug Sensitivity in Cancer (GDSC) the drug sensitivity of about 1000 cancer cells is the drug sensitivity of data (http://www.cancerrxgene.org). The Spearman correlation analysis is used to calculate the correlation between drug sensitivity and CRRS, taking half-maximal inhibitory concentration (IC50) as the drug response index in cancer cell lines, and considering p < 0.05 and coef > 0.2 was a significant correlation. The same method is also done in Cancer Cell Line Encyclopedia (CCLE) database (https://depmap.org/portal/download/).

### Transcription factor regulatory network, RNA velocity, and cells communication

RcisTarget database of humans (https://resources.aertslab.org/cistarget/) and R package “SCENIC” are used for transcription factors regulating network building [27]. AUCell algorithm is utilized to assess transcription factor activation and regulatory modules based on the connection-specific index. Besides, RNA velocity analysis of tumor cells is calculated by package “monocle3” [28]. Different states of OC cells are figured to reveal their internal transformation. Communication between immunocytes and OC cells is analyzed using the R package “Celltalker” and “Cellchat”, and differential ligand-receptor pairs are identified [29].

### Human OC tissue specimens and immunohistochemical staining

In our work, the clinical patient tissue microarray contained 8 40 epithelial ovarian cancer from department of Gynecologic Oncology, Hunan Cancer Hospital. All tissue specimens are confirmed by pathologist diagnosis and bedded in paraffin for immunohistochemistry (IHC). IHC staining and score criteria are described as previous research [30]. The primary antibody used was anti-KIF26B (dilution 1:200, 17422-1-AP, Proteintech). The informed consent is given to patients before this research.

### Quantitative real-time PCR and small interfering RNA

Total mRNA was extracted from OC cells using Trizol reagent (Takara) following the operating protocol. Reference gene GAPDH is used to normalization. Primer sets used for KIF26B and GAPDH RNA examination are as follows: KIF26B forward 5ʹ- TTTGCGCCACTCACCTGAA -3ʹ, KIF26B reverse 5ʹ- GGCGTCGTAGTGCTCACTG -3ʹ; 18 s forward 5ʹ-TGCGAGTACTCAACACCAACA-3ʹ,18 s reverse 5ʹ- GCATATCTTCGGCCCACA-3ʹ. The formula RQ = 2 − ΔCT is used to calculate gene expression levels.

The siRNAs against KIF26B were purchased from Gene Pharma (Shanghai, China). Transfection according to the manufacturer’s protocols uses Lipofectamine 3000. For KIF26B siRNA: siKIF26B-1: 5- GGACAACCGCUGUGACAUUTT-3, siKIF26B-2: 5-CAUCGAGAGUCUUGAGGAUTT-3.

### Cell viability, colony formation, and Edu stain assay

The CCK-8 cell proliferation, colony formation, and apoptosis assay were performed as previously described [30].

### Statistical analysis

Statistical analyses are executed utilizing R software (version 4.1.0) and RStudio (version 2022.07.2). The SPSS 19.0 and GraphPad Prism 8.0 software is used for statistical analysis. Student’s t-test is used to analyze two groups of data. All statistical *p* values are two-sided and less than 0.05 is considered statistically significant. All methods were carried out in accordance with relevant guidelines and regulations.

## Results

### Expression change of CRGs and consensus clustering analysis of ovarian cancer patients

**Fig. 1:**
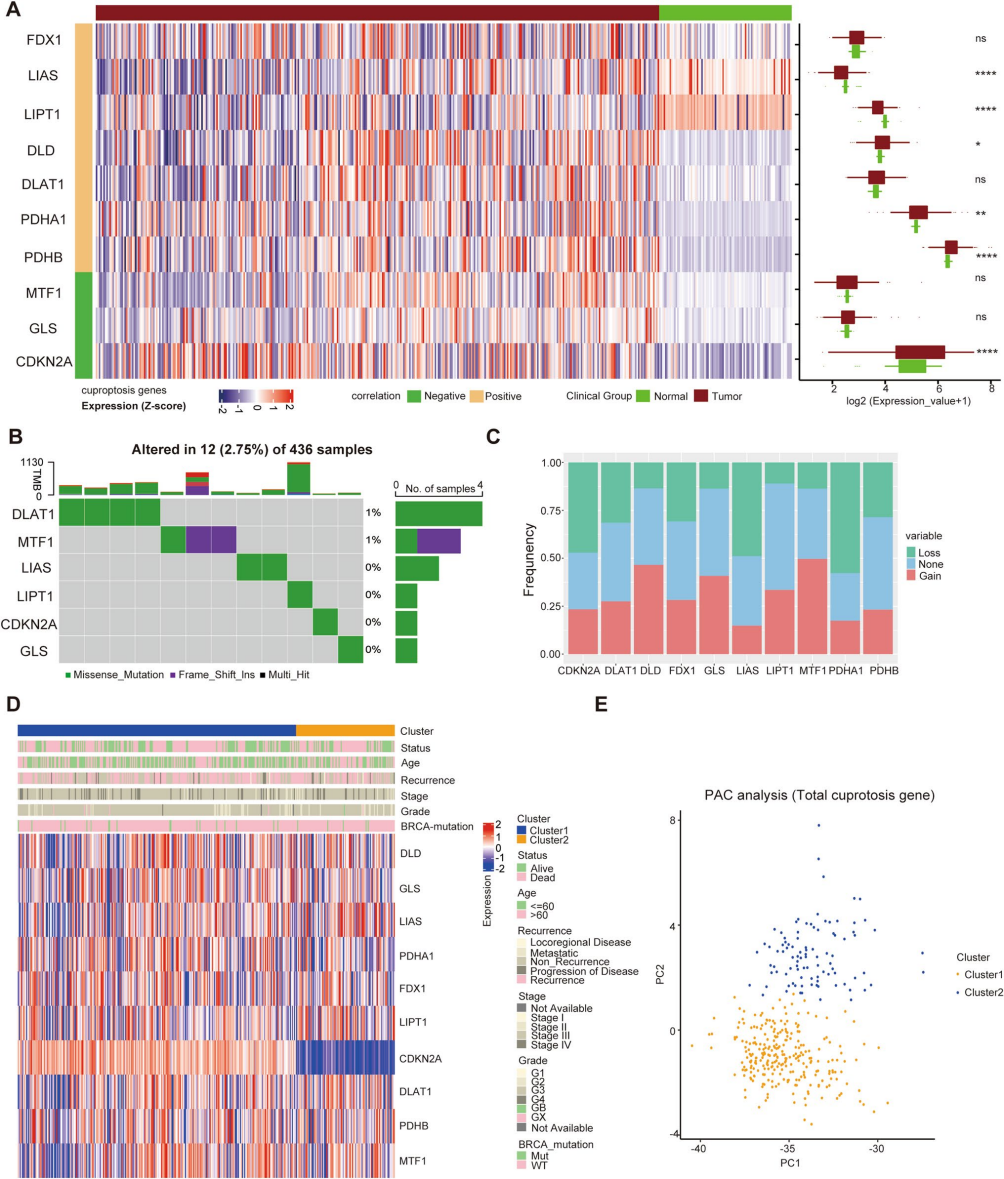
Expression and genetic alteration of CRGs in OC: **A** the expression of 10 CRGs in OC and normal ovary tissues; **B**, **C** the CNV and mutation frequencies and classifications of 10 CRGs in OC patients from TCGA cohort. **D** Unsupervised clustering of 10 cuproptosis-related genes in TCGA OC cohort. Cuproptosis cluster, live status, age, recurrence, stage, grade, and BRCA mutation were utilized as patient annotations. **E** PCA analysis of two cuproptosis subtypes in TCGA OC cohort. *p < 0.01, ***p < 0.001

In this work, 10 classical CRGs were confirmed in the literature. Based on the TCGA and Genotype-Tissue Expression (GTEx) datasets, the heatmap revealed the differential mRNA expression of 10 cuproptosis-related genes (CRGs) in OC and normal samples. The results indicated that 6 genes (LIAS, LIPT1, DLD, PDHA1, PDHB, and CDKN2K) had significant differential expression (Fig. 1A). Our further summary analysis of the incidence of somatic mutations in these 10 CRGs revealed that 12 patients (2.75%) in the OC cohort had CRG mutations (Fig. 1B). Among them, the mutation frequency of DLAT was the highest, followed by MTF1, LIAS, LIPT1, CDKN2A, and GLS, while the other 4 CRGs had no mutation. Subsequently, we explored somatic copy number changes in these CRGs and detected common copy number changes in all 10 CRGs. Among them, DLD, MTF1, GLS, and LIPT1 had a high degree of copy number gain, while LIAS and PDHA1 had copy number loss (Fig. 1C). The locations of the copy number variations (CNVs) in CRGs on their respective chromosomes are shown in Fig S1A.

To further illuminate the clinical features or biological behaviors of these CRGs, we performed an unsupervised clustering algorithm and divided the TCGA OC cohort into 2 groups (k = 2) based on the expression profiles of 10 CRGs; there were 268 patients in cuproptosis-related Cluster 1 and 95 patients in cuproptosis-related Cluster 2 (Fig S1A, B, C). Cluster 1 had a survival advantage over Cluster 2 according to Kaplan–Meier survival analysis (log-rank test, p = 0.048; Fig S1D). We also explored the clinicopathological characteristics of these patients. The heatmap showed no significant difference in *BRCA* mutation and recurrence between the 2 clusters (Fig. 1D). Principal component analysis (PCA) demonstrated distinct separation in the cuproptosis transcriptional profiles between the 2 clusters (Fig. 1E).

### Identification immune infiltration characteristics and differentially expressed genes between different clusters

**Fig. 2:**
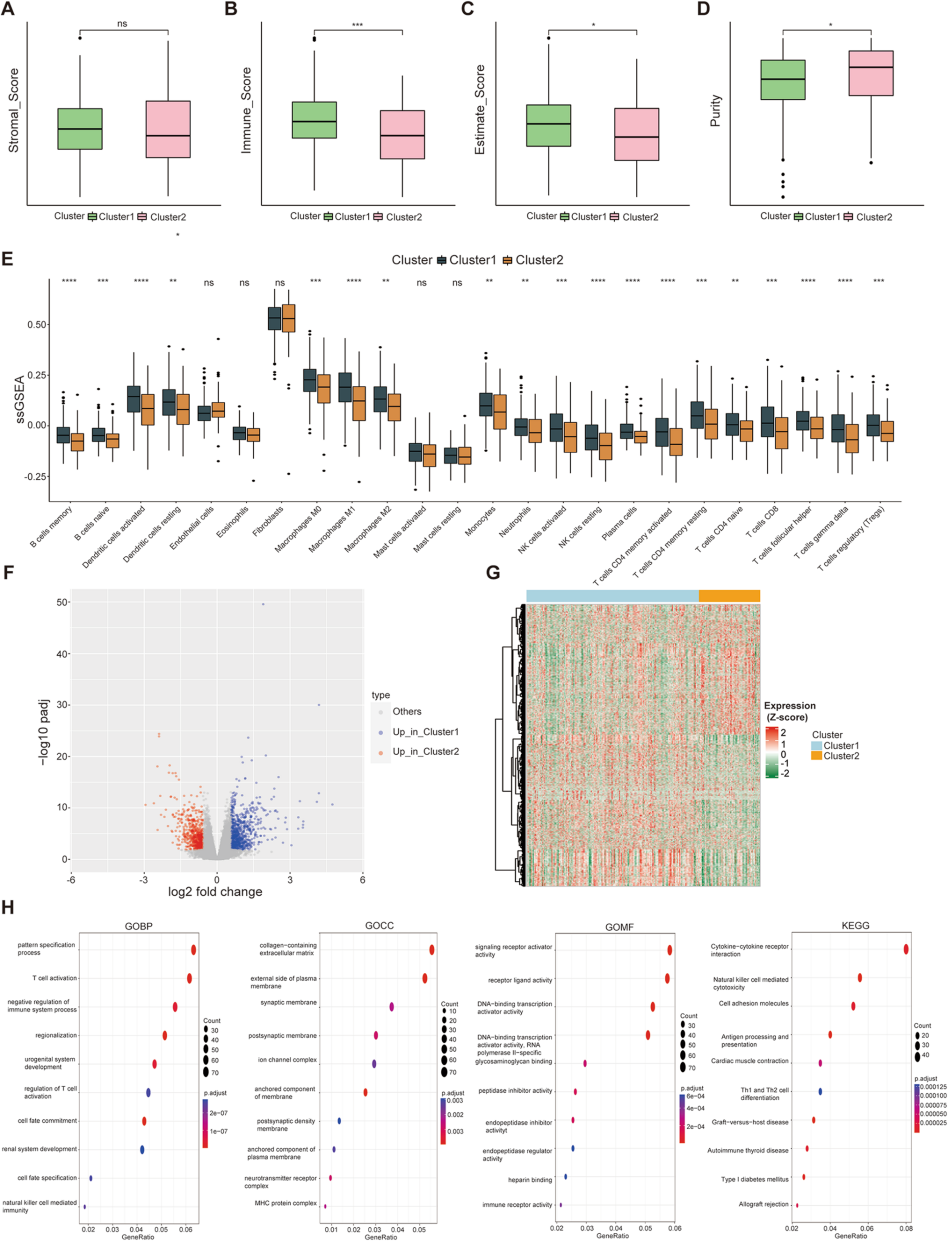
Identification immune infiltration characteristics and DEGs in the two clusters. **A**–**D** Stromal score, immune score, estimate score, and purity between the OC clusters. **E** The GSA scores for each TME infiltrating cell in the two OC clusters. The line in the box represents the median value. **F**, **G** The volcano plot and heat map show the unsupervised clustering of DEGs in the TCGA OC cohort. **H** GO and KEGG enrichment analysis of DEGs. GO, Gene Ontology. KEGG, Kyoto Encyclopedia of Genes and Genomes. DEGs differentially expressed genes. TME, tumor microenvironment; ssGSEA, single sample gene set enrichment analysis. *p < 0.01, ***p < 0.001

As shown in Fig. 2A-D, the ESTIMATE algorithm was used to estimate the ratio of the immune matrix component of the tumor microenvironment (TME) based on the TCGA expression profiles; TME features were reflected by the immune score, stromal score, and estimate score, which are positively correlated with the infiltration level of immune cells and the content of matrix components [31]. The results showed that the immune score and estimate score was significantly higher in Cluster 1 than in Cluster 2, while no significant differences were shown between the two clusters in terms of stromal score. We further explored the relationship between clusters and immune cell infiltration established in the TCGA cohort by using the ssGSEA algorithm (Fig. 2E). The great majority of immune cells exhibited significantly higher infiltration in Cluster 1 samples than in Cluster 2 samples; these cells included memory B cells, dendritic cells, macrophages, CD4 T cells, CD8 T cells, natural killer cells, follicular helper T cells, and Tregs.

To further investigate the conceivable biological processes of the cuproptosis-related clusters in the OC cohort, we utilized the R package “DEseq2” to identify the differentially expressed genes (DEGs) among the 2 clusters; 783 genes were highly expressed in Cluster 1 and 595 genes were highly expressed in Cluster 2 (Fig. 2F, G). GO enrichment analysis of the DEGs demonstrated that these DEGs were mainly related to some immune and carcinogenesis biological processes, such as T-cell activation, negative regulation of immune system process, regulation of T cell, signaling receptor activator activity, DNA − binding transcription activator activity, and immune receptor activity. Moreover, in the KEGG pathway enrichment analysis, these DEGs were mainly related to cytokine − cytokine receptor interaction, natural killer cell-mediated cytotoxicity, Th1 and Th2 cell differentiation, and cell adhesion molecules (Fig. 2H).

### The cuproptosis-related scoring model exhibits great prognostic prediction ability

**Fig. 3:**
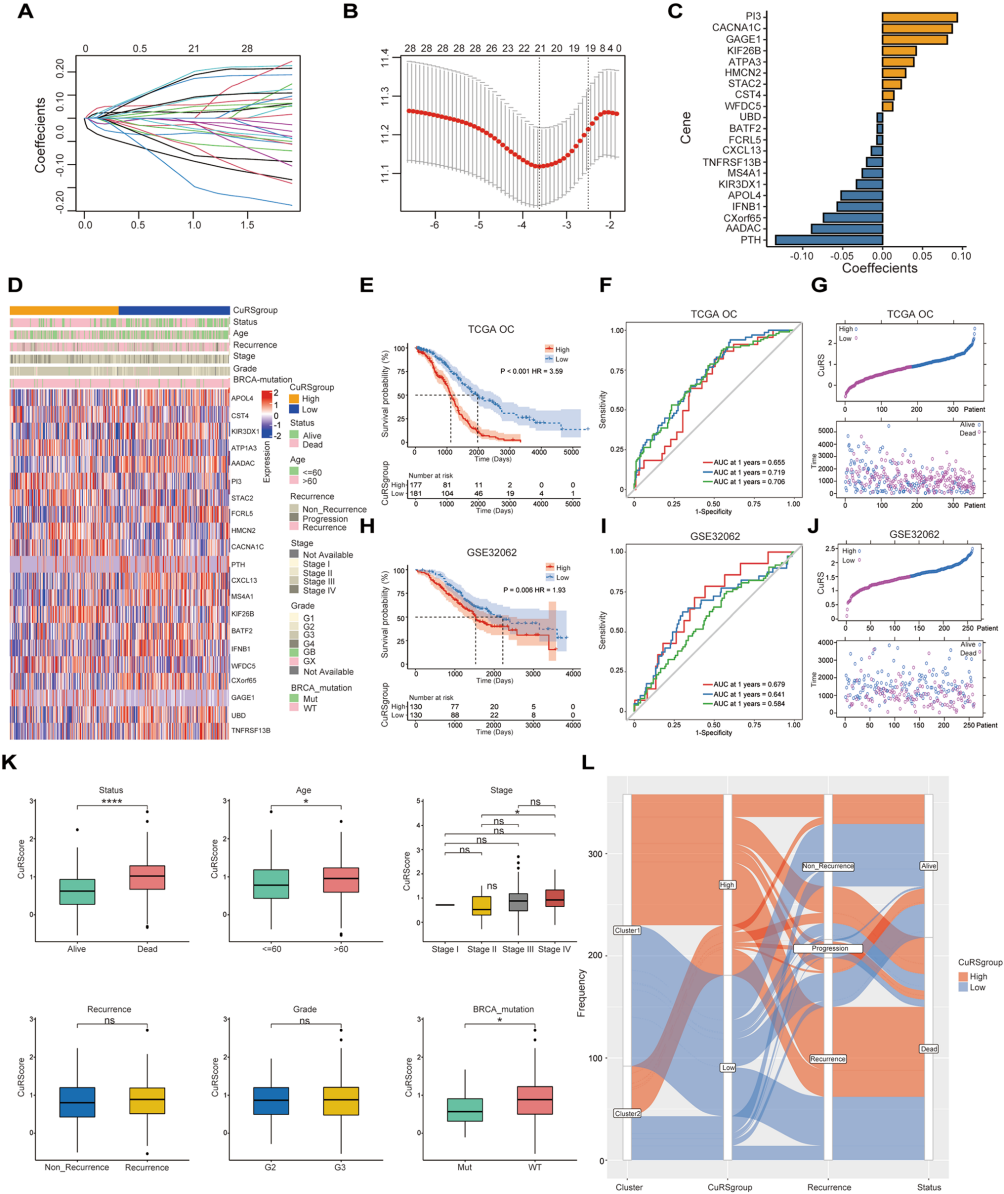
Construction of the CuRS model (**A**, **B**) Cvfit and lambda curves illustrating the LASSO regression model produced using tenfold cross-validation. **C** Coefficients of the 21 prognostic molecules in the Cox regression model. **D** The heatmap representing differences in the clinical information and genes expression between the high and low GuRS clusters. **E** Kaplan–Meier plots of OS for the two GuAS clusters in the TCGA data. **F** 1-, 2-, and 3-year ROC of GuAS prognostic performance in the TCGA data. **G** Ranked dot and scatter plots for the relationship between GuAS and prognosis status in the TCGA data. **H**, **I**, **J** Kaplan–Meier plots, ROC curve, and scatter plots of GuAS prognostic performance in GSE32062 data. **K** GuRScore differences among different clinical features in the TCGA data. **L** An alluvial diagram of the distribution of cuproptosis cluster, gene cluster in two risk groups, as well as survival outcomes.*p < 0.01, ***p < 0.001

Using univariate Cox regression analysis, 33 prognostic cuproptosis-related genes were identified among the 1378 DEGs (Fig S2A). The cuproptosis scoring model was constructed depending on these prognostically significant DEGs. Least absolute shrinkage and selection operator (LASSO) Cox regression was applied to screen out the 33 prognostic DEGs to determine the optimal penalty parameter λ, and 21 genes were finally selected. A cuproptosis-related prognostic model was then established on the basis of the expression profiles of the 21 prognostic genes, and this model was named the CuRScore model (Fig. 3A–C).

Based on the median CuRScore, the samples in the TCGA and GSE32062 OC cohorts were further stratified into high-risk or low-risk subgroups (Fig. 3D). Patients in the high-risk subgroup showed substantially shorter overall survival than those in the low-risk subgroup according to the Kaplan‒Meier method in both cohorts (Fig. 3E, H). The area under the curve (AUC) was generated to compare prognostic ability within different models, and analysis indicated that CuRS possessed relatively high AUC values for 1-, 2-, and 3-year overall survival (0.655, 0.719, and 0.706, respectively) (Fig. 3F, G). Additionally, the AUCs for risk variables were 0.679, 0.641, and 0.584 in the GSE32062 OC cohort, which demonstrated a relatively high excellent predictive ability for OC patients (Fig. 3I, J). Moreover, we found that older age (> 60), advanced stage (stage III), death, and *BRCA* mutation were closely correlated with a high CuRS, whereas grade and recurrence revealed no relationship with CuRS (Fig. 3K). Moreover, considering the clinicopathological characteristics, CuRS was confirmed as an independent predictive factor for OC in the TCGA and GSE32062 cohorts by univariate and multivariate Cox regression analyses (Fig S3 A–D). Figure 3L illustrates the distribution of patients in the two cuproptosis clusters and two CuRScore subgroups. The majority of patients in cuproptosis cluster 2 were also in the low-risk group, which had a good prognosis, similarly, cluster 1 with high-risk score patients had the worst survival.

### Analysis of CuRS model using single‑cell RNA sequencing

**Fig. 4:**
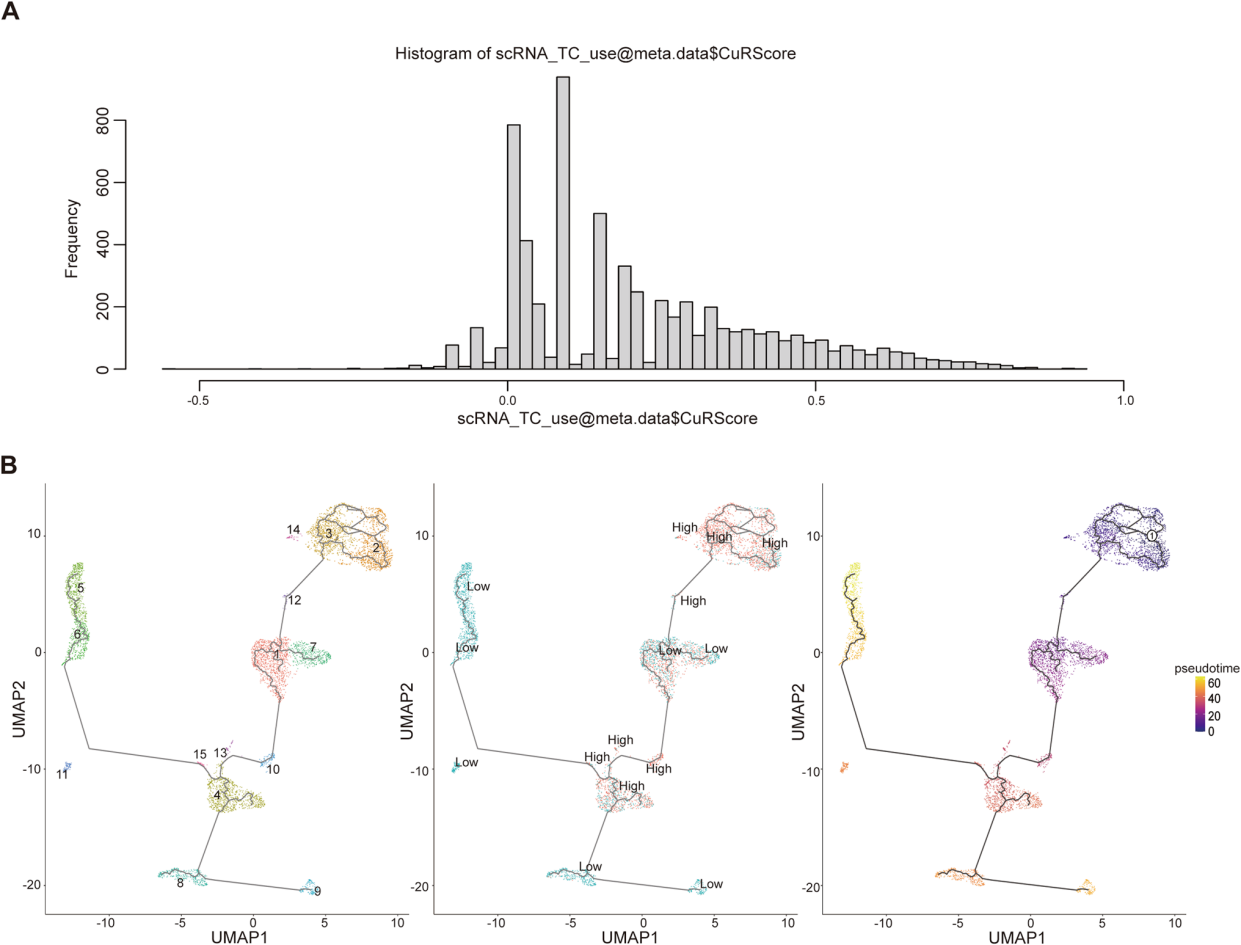
Single-cell clustering analysis and pseudotime analysis in OC cohort. **A** The frequency distribution of the CuRscoring in OC cells. **B** The pseudotime trajectory in OC cells

After quality control, we utilized signal cell RNA-seq data (GSE17368 and GSE15893) to acquire gene expression profiles of at least the 21 signature genes for 6426 cells from 7 OC samples. Based on these malignant epithelial cells, a total of 15 clusters were identified, and 8 subtypes were confirmed based on CNVs (Fig S4A). Moreover, according to the median CuRS value, the 15 clusters of single cells were classified into high and low groups (Fig S4B). The allocation of the CuRScore was assessed and mainly existed between − 0.1 and 1 (Fig. 4A). Sorting of these cells according to the gene expression of each cell enabled further inference of the cell development pathway by the Monocle 3 algorithm. Consequently, a lineage from low CuRS OC cells to high CuRS OC cells was identified (Fig. 4B), and this lineage was consistent with the evolutionary pathway of the CuRS cluster. Copy number variation is a gradual development process, and according to the chronological order, the tumor cells that are closest to the copy number profile of normal cells should appear at an earlier time. As shown in Fig. 4, the CuRScore is positively correlated with tumor cell progression. Moreover, cluster 4, as the subtype with the lowest CNV frequency, was more likely than other clusters to be normal cells at represent the beginning of the progression of carcinogenesis (Fig S5A).

### Transcription factor differentially activated in high and low CuRS groups

**Fig. 5:**
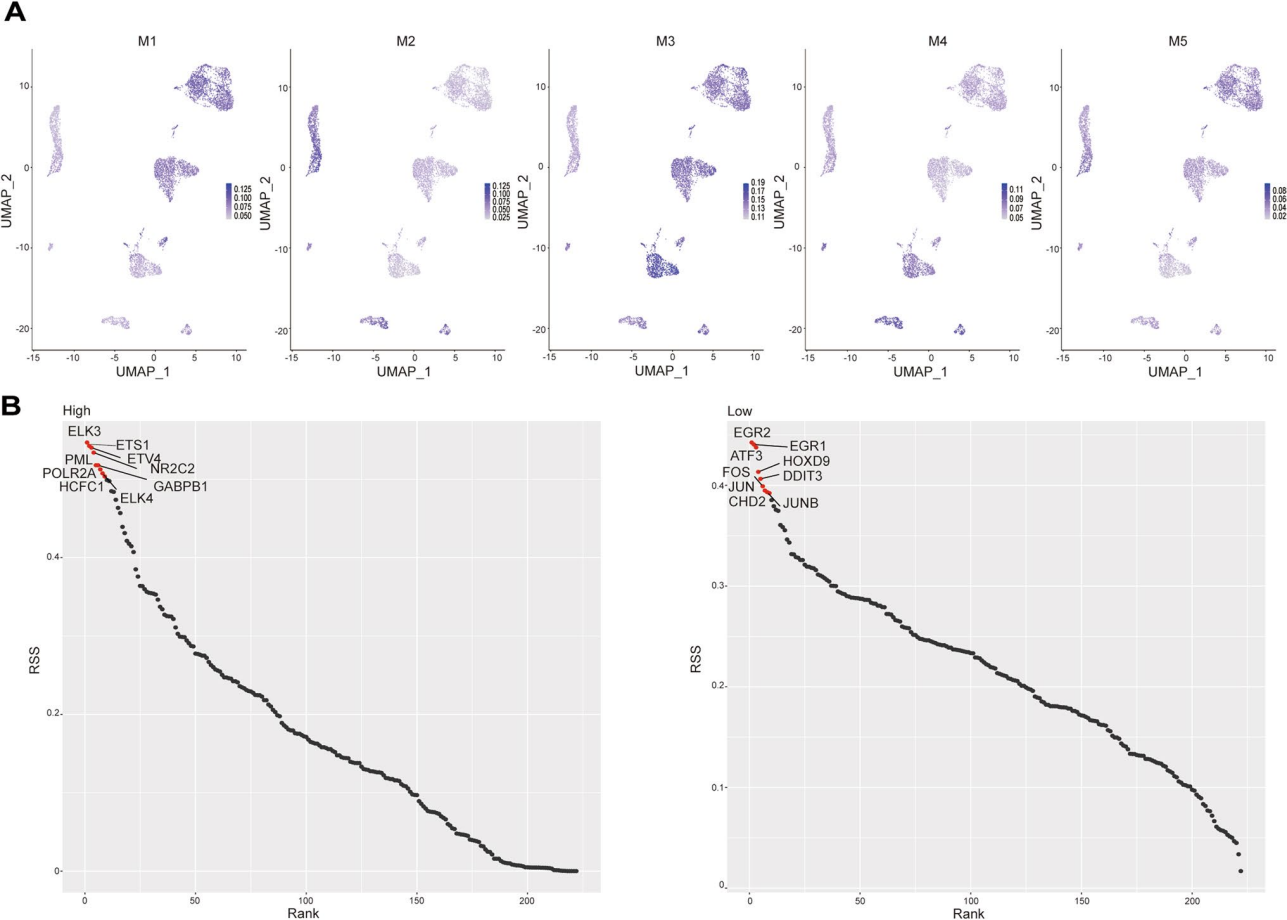
Transcription factor activation difference in two GuAS cluster cells. **A** The correlation between the scoring system and those modules relies on RNA velocity. **B** Top 10 differential activated TFs in the two GuRS clusters respectively

Furthermore, we investigated the activation of transcription factors in two CuRS groups of OC cells. Because transcription factors mutually cross-regulate the expression of some genes, we grouped them into different modules (M1, M2, M3, M4, M5) (Fig S6A). Each module represented a group of collaborative transcription factors. The regulon activity score (RAS) of the respective module was computed according to the Connection Specificity Index defined in a previous study [32]. It appeared that M3 had a higher RAS than other modules in both CuRS groups, while the RAS of M5 was relatively low. Moreover, the RAS of M2 was negatively correlated with CuRS. There was no available expression diversity in the RAS of M4 and M1 between the high and low CuRS groups (Fig. 5A). The scores of the activity of regulons of these modules were classified with the AUCell algorithm and then shown by t-SNE plots. The M2 module was more activated in the low CuRS group (Fig S6B). We also analyzed the top 10 transcription factors in the high and low CuRS groups using the regulon specificity score (RSS).

Then, we selected the top 10 transcription factors from the high (ELK3, ETS1, ETV4, NR2C2, PML, GABPB1, POLR2A, ELK4, HCFC1, and ETS2) and low (EGR3, EGR1, ATF3, HOXD9, DDIT3, FOS, JUN, JUNB, CHD2, and ELF3) CuAS groups for further analysis (Fig. 5B). The figure S6C, D shows the top 5 transcription factors enrichment distribution map in the high and low CuRS groups. Meanwhile, we performed GO and KEGG pathway enrichment analyses on the corresponding target genes based on the top 5 differential transcription factors. The transcription factors of high CuRS were significantly enriched in immune-related pathways, while TFs of cells with low CuRS were significantly enriched in pathways such as apoptosis. Moreover, tumor transcriptional dysregulation pathways were significantly enriched in both high and low CuRS cells (Fig S7).

### Immune related pathways selectively activated in high and low CuRS groups

**Fig. 6:**
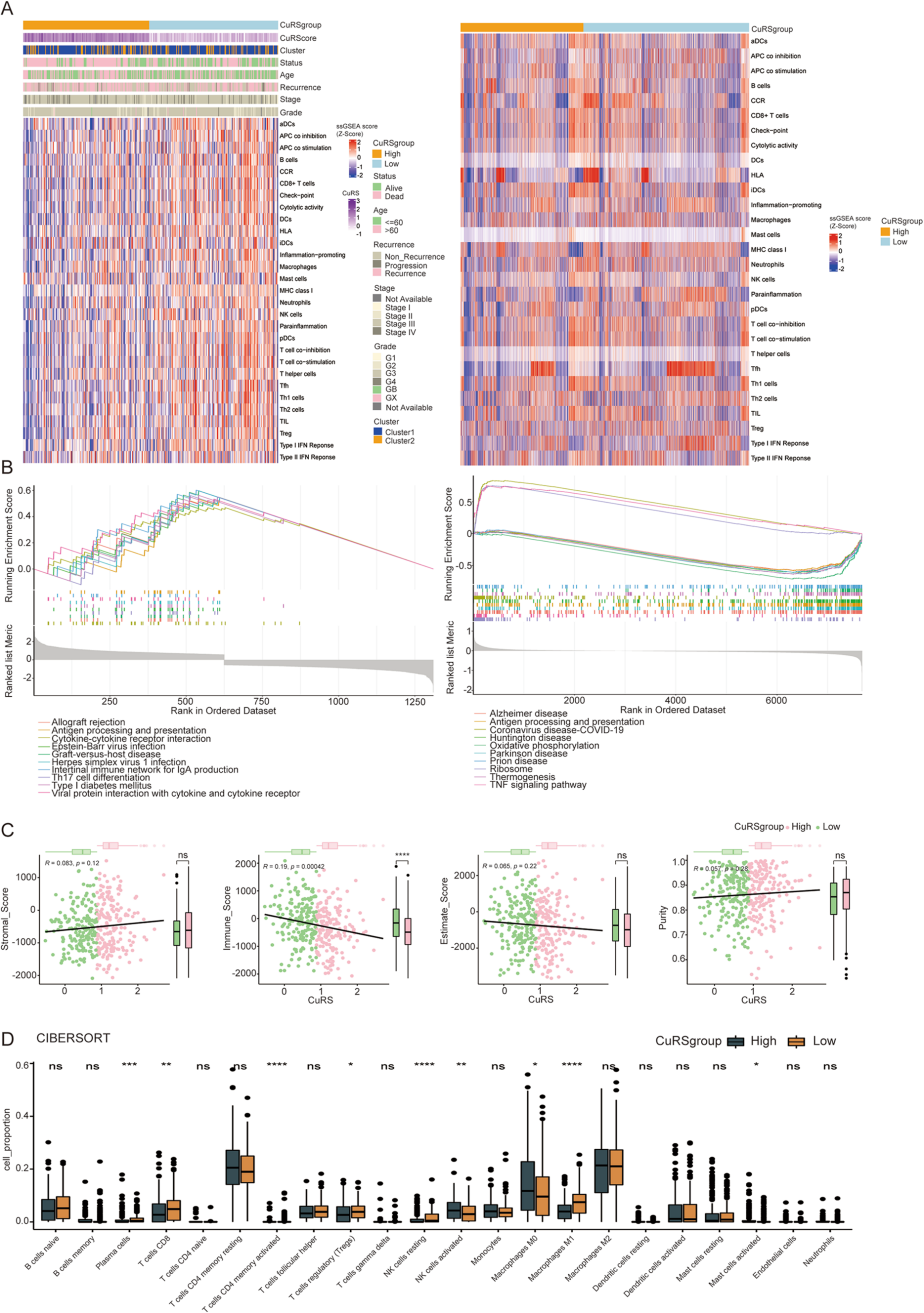
Immune-related pathway analysis rely on bulk RNA-seq and scRNA-seq data in OC. **A** The heatmap represents differences in the clinical information and immune-related pathways in bulk RNA-seq analysis (left) and scRNA-seq analysis (right). **B** KEGG enrichment analysis based on the GSVA algorithm in bulk RNA-seq (left) and scRNA-seq analysis (right). **C** Stromal score, immune score, estimate score, and purity between the high and low GuAS clusters. **D** The TME infiltrating cell base on the CIBERSORT algorithm in the two GuAS clusters. The line in the box represents the median value. *p < 0.05, **p < 0.01, ***p < 0.001

We further performed an enrichment analysis of 29 immune-related pathways using the GSVA algorithm based on bulk RNA-seq data. Correlation analysis demonstrated that the CuRS with prognostic signatures was connected with most of the 29 immune-related pathways, and similar results were found in the scRNA-seq data (Fig. 6A). The KEGG enrichment analysis based on the GSVA analysis on OC patients from bulk RNA-seq data demonstrated that the high CuRS cluster was significantly related to the activation of immune-related pathways, including antigen processing and presentation, the intestinal immune network for IgA production, and Th17 cell differentiation. In addition, according to the scRNA-seq data, these results showed that the high CuRS cluster was significantly associated with the oxidative phosphorylation metabolic pathway, ribosome, and TNF signaling pathway (Fig. 6B). Furthermore, the proportion of infiltrating immune cells was analyzed using the CIBERSORT algorithm and was found to be significantly different between the high and low CuRS clusters for cells such as M0 and M1 macrophages, CD4 T cells, CD8 T cells, activated NK cells, and activated mast cells (Fig. 6C, D).

### Novel ligand–receptors pairs between immunocytes and the CuRS model

**Fig. 7:**
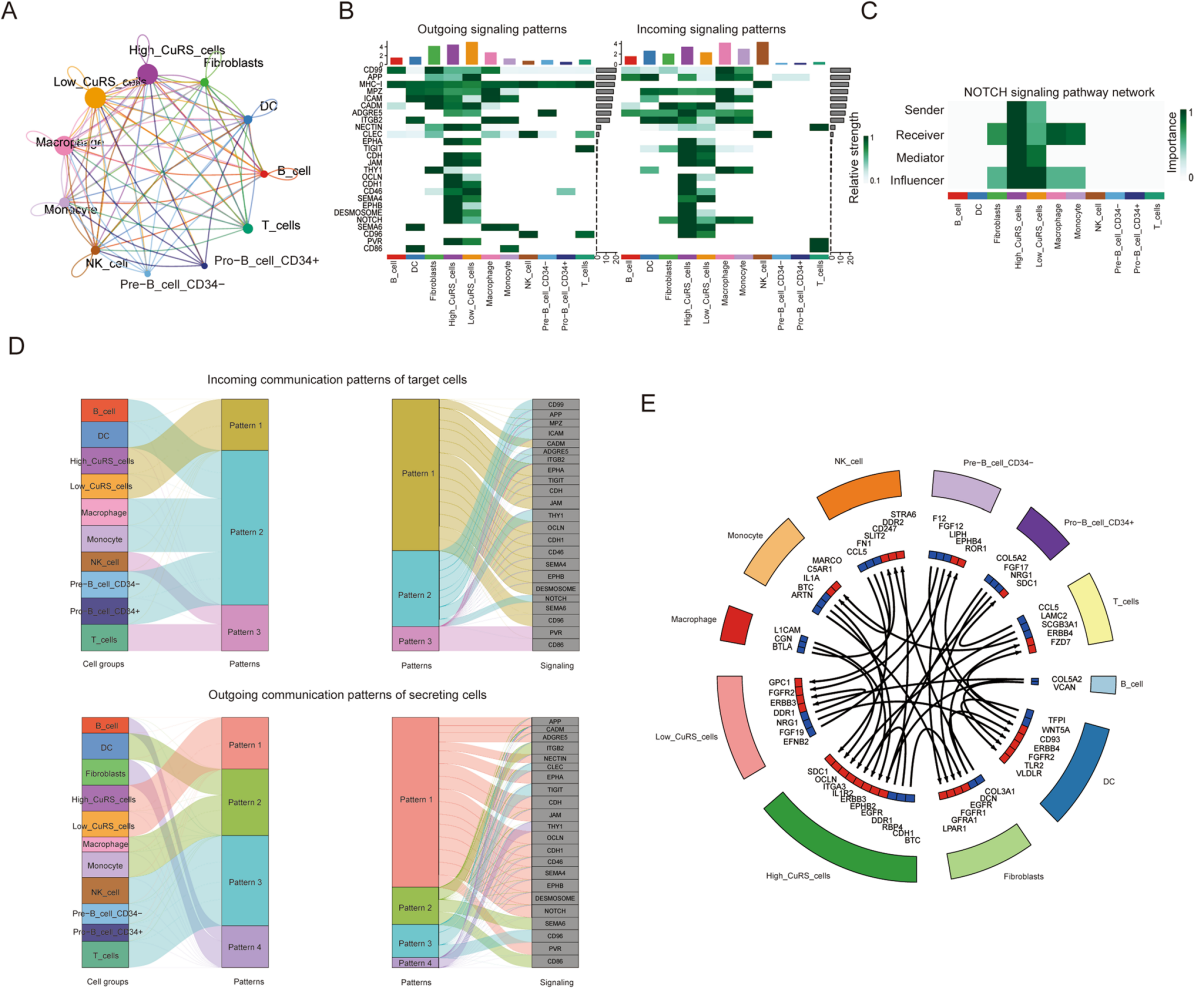
Novel ligand–receptor pairs difference between high and low CuRS clusters. **A** The cross-talk intercommunication among CuRS OC cells and different immunocytes. **B** The correlation between CuRS groups or immunocytes and different signaling pathways. **C** NOTCH signaling pathway network. **D** Incoming and outcoming communication patterns of target and secreting cells. **E** ligand–receptor pairs among different cells

Then, we analyzed the intercommunication patterns between OC cells and immunocytes by using the CellChat algorithm based on single-cell RNA-seq analysis (Fig. 7A). As illustrated, there were specific communication patterns for both incoming and outgoing signaling in high and low CuRS cells, where the SEMA4 pathway had significant communication intensity differences between the high and low CuRS groups in terms of outgoing signaling patterns. Additionally, regarding incoming signaling patterns, the SELL pathway had a significant difference in communication intensity (Fig. 7B). The Notch pathway exhibited a significant difference in sender, receiver, and influencer patterns (Fig. 7C). Figure 7D illustrates the communication pattern distribution of the signaling pathways in OC cells and immunocyte cells. Furthermore, we utilized the CellTaker algorithm to identify ligand‒receptor pairs between immunocytes and CuRS cells. As shown in Fig. 7E, OC cells with high CuRS communicate with macrophages, T cells, CD34 + B cells, fibroblasts, and monocytes more actively than OC cells with low CuRS, which may explain the immune landscape difference.

### Chemotherapy differential sensibility in high and low CuRS groups

**Fig. 8:**
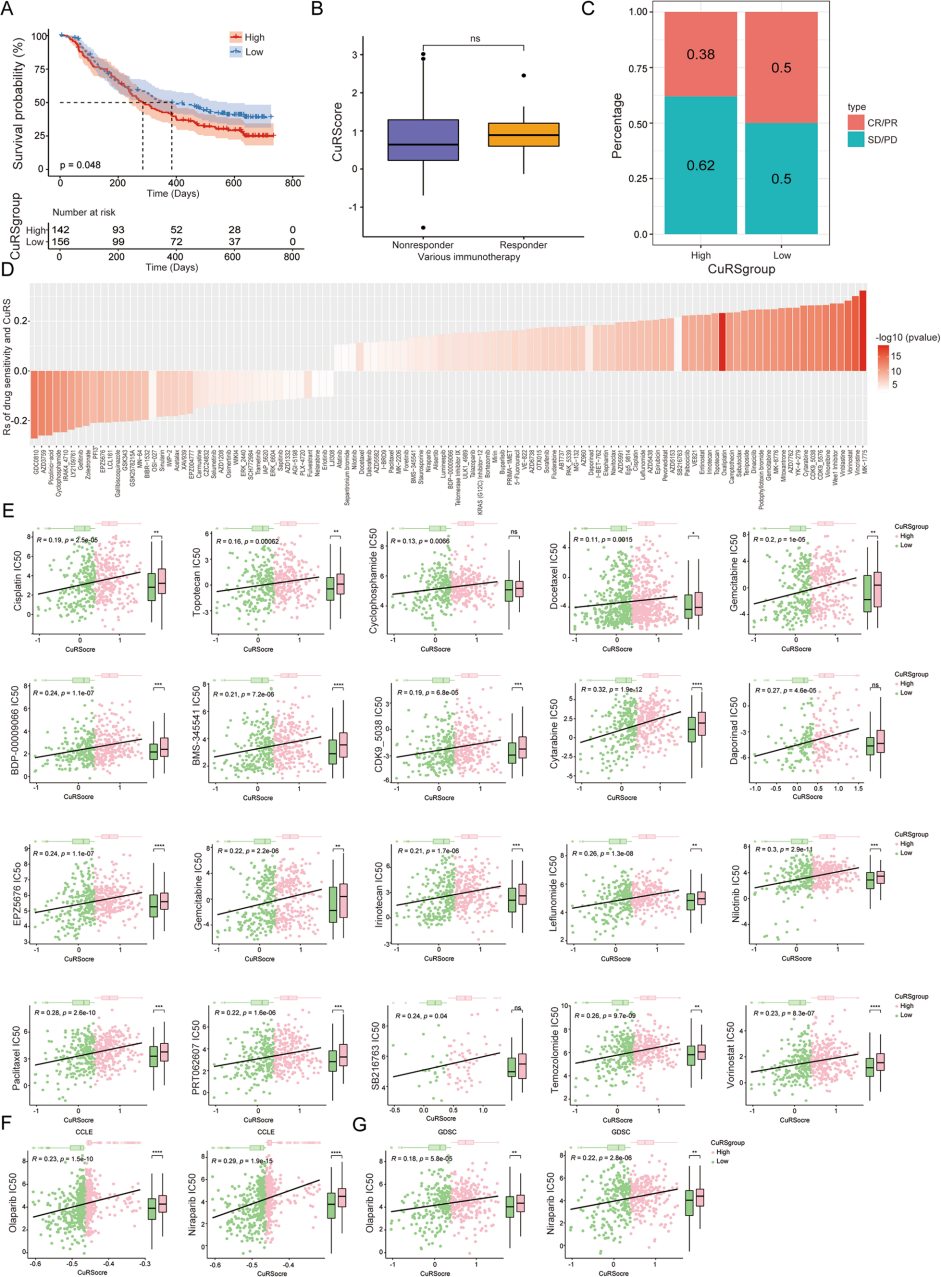
The correlation between CuRS and drugs sensitivity. **A** Kaplan–Meier curve of OS with high and low GuAS clusters for IMvigor210 cohort. **B**, **C** Boxplot and Bar’s graph shows the therapeutic response [complete response (CR)/partial response (PR) and stable disease (SD)/progressive disease (PD)] to immunotherapy in the two GuAS clusters in IMvigor 210 cohort. **D** The relationship of drug sensitivity and CuRS score. **E** Drugs characterized by IC50 positively correlated with CuRS score in the CCLE database. **F**, **G** PARP inhibitors sensitivity positively correlated with CuRS score in the CCLE and GDSC database. ***p < 0.001

To further explore the correlation between the CuRS subgroups and the effectiveness and sensitivity of chemotherapy, the OC chemotherapy cohort with considerable absolute clinical data was investigated based on the imvigor210 database. Patients in the high CuRS group had a substantially worse prognosis in the imvigor210 OC cohort (Fig. 8A). However, there were no significant differences in the chemotherapy drug response rate, CR/PR rate, or SD/PD rate between the high and low CuRS groups (Fig. 8B, C). These results suggested that the CuRS could be utilized to predict the efficacy of OC patient chemotherapy survival. Additionally, we analyzed the differences in chemotherapy and targeted therapy drug resistance between the high and low CuRS groups (Fig. 8D). Based on the CCLE database, the IC50 values of cisplatin, topotecan, cyclophosphamide, docetaxel, and 5-fluorouracil were higher in patients with high CuRS, and there was a significant positive correlation between IC50 and CuRS (Fig. 8E). Remarkably, similar results were obtained from the GDSC database analysis. In the GDSC database, we identified 172 kinds of chemotherapy drugs with a significant positive correlation between IC50 and CuRS subgroups. Among them, 13 kinds of chemotherapy drugs had correlation coefficients greater than 0.3, and no chemotherapy drug IC50 had a significant negative correlation with CuRS (Fig S8). We also focused on the relationship between CuRS subgroups and resistance to the PARP inhibitors olaparib and niraparib. In both the CCLE database and GDSC database, in patients with high CuRS, the IC50 values of olaparib and niraparib were significantly increased (Fig. 8F, G). In conclusion, these results suggest that drug IC50 has an excellent positive correlation with CuRS and CuRS was associated with chemotherapy and targeted therapy drug resistance.

### Knockdown of KIF26B increased the sensitivity of OC cells to cuproptosis agonists

**Fig. 9:**
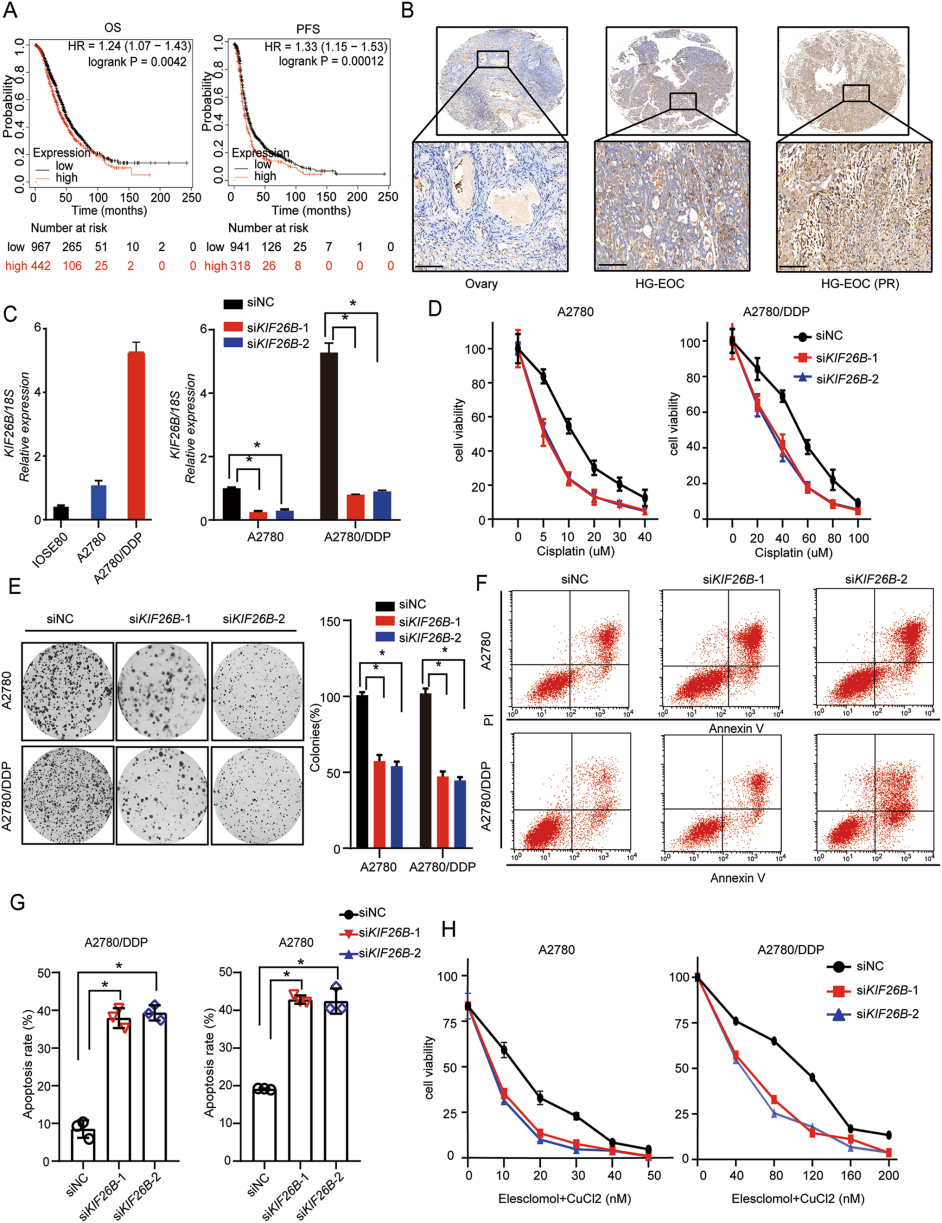
KIF26B is related to copper ionophore-induced cell death. **A** Kaplan–Meier analysis of the OS and PFS of OC platinum chemotherapy patients with KIF26B high or low expression level. **B** Representative IHC images showing KIF26B expression in normal ovarian epithelial tissues, platinum-sensitive, and platinum-resistant OC specimens. **C** the expression level in IOSE80, A2780, A2780/DDP and after KIF26B knockdown. **D** CCK-8 assay analyzes cell viability after KIF26B knockdown treatment with cisplatin. **E** Colony formation and quantification analysis after KIF26B knockdown treatment with cisplatin. **F**, **G** Representative apoptosis assay and quantification analysis after KIF26B knockdown treatment with cisplatin. **H** CCK-8 assay analyzes cell viability after KIF26B knockdown treatment with elesclomol-CuCl2. *p < 0.05

According to the above results, we further explored whether the expression of cuproptosis model related genes contributed to platinum resistance in OC. The online kmplot database (http://kmplot.com/analysis/) analysis results suggested that high expression of KIF26B, which among cuproptosis-related genes, predicted poorer prognosis in ovarian cancer patients treated with cisplatin (Fig. 9A). The IHC showed that the protein level of KIF26B in ovarian cancer was significantly higher than that in normal ovarian epithelial tissues. Meanwhile, the level of KIF26B protein in platinum-resistant OC samples was significantly higher than that in platinum-sensitive OC samples (Fig. 9B). To clarify the biological function of KIF26B, we constructed a cisplatin resistant ovarian cancer cell line A2780 (A2780/DDP). The expression levels of KIF26B in OC cell lines and the efficiency of knockdown KIF26B were confirmed by quantitative real-time PCR (Fig. 9C). The CCK-8 assay and clonogenic assay showed that knockdown of KIF26B expression significantly reduced ovarian cancer cell proliferation (Fig. 9D, E). In line with these findings, the apoptosis assay was utilized to validate the above results (Fig. 9F, G). Moreover, under the treatment of Elesclomol-CuCl2, the CCK-8 assay results revealed that the sensitivity of OC cells to cuproptosis agonist was increased after knockdown of KIF26B (Fig. 9H). These results thus suggested that the knockdown of KIF26B facilities cuproptosis in OC cells.

## Discussion

Copper is a valuable coenzyme in many physiological activities, such as the oxidative stress response, lipid metabolism, and mitochondrial respiration [33]. Cuproptosis, an unconventional mechanism of cell death, is playing an increasingly important role in promoting tumor progression, recurrence, TME, and chemotherapy response [34, 35]. The mechanisms of copper-induced tumor cell death include induction of reactive oxygen species production, decreasing the expression of signaling pathways, and overcoming chemoresistance, but its effects on OC remain poorly understood. In ovarian cancer, previous studies showed that CDKN2A, the mutations of which led to loss of growth control in ovarian cancer cells, is related to the cell cuproptosis sensitivity [9, 36], indicating that cuproptosis plays essential role in ovarian cancer.

Infiltration of immune cells was validated to be associated with cancer cell proliferation, invasion, and metastasis in multiple cancers [37–40]. Some immune cells were differentially infiltrated in the CuRS subgroups. While the high-risk group had more M0 macrophages, resting memory CD4 T cells, activated NK cells, and activated mast cells, the low-risk group had more CD8 T cells and M1 macrophages. The ssGSEA algorithm results also revealed that patients in the low-risk group had higher immune activity. Tumor-associated macrophages (TAMs) are the key regulatory agent of treatment responses in the TME [41]. M2 macrophages, as a major subtype of macrophages, are conducive to tumor cell growth, invasion, and angiogenesis, and these cells are associated with a poor prognosis in many cancers [42]. In contrast, a high level of M1 macrophages is related to antigen presentation and indicates antitumor activity in patients with ovarian cancer [43]. Many studies have shown that a high level of T-cell infiltration, particularly cytotoxic CD8 T cell infiltration, indicates a favorable prognosis, and our findings support these conclusions [44]. According to the ESTIMATE algorithm, a low-risk group had higher immune scores than a high-risk group. As previously reported, the higher TMB is associated with better prognosis in OC patients [45], which is consistent with the findings in this study. These results suggested that our model can accurately predict the prognosis and immune status of OC patients and develop personalized immunotherapeutic strategies.

As previously shown, no studies have revealed the cellular relationship between cuproptosis and the progression of OC or the immune landscape. By integrating bulk and single-cell RNA sequencing, we first systematically analyzed the heterogeneity of OC. Based on the cuproptosis genes, we classified 15 clusters and 8 subtypes of OC cells using the Seurat package and copy number variation (CNV) profile, and these clusters were visualized by using the UMAP algorithm. Pseudotime trajectory analysis via the Monocle 3 algorithm also illustrated the time-dependent evolution of OC cells among these clusters. In our study, we show that the differentiation and progression of OC cells is classified along with increasing CuRS, which demonstrates that the CuRS scoring model can be used to evaluate tumorigenesis.

The development of chemotherapy drug resistance is an important cause of recurrence and death in patients, but there is no effective treatment [46]. Copper transporters mediate resistance of OC cells to platinum-based drugs, and may predict platinum sensitivity and prognosis of OV patients. Prevent study showed that cisplatin chemotherapy resistance by glutathione-resistant copper-based nanomedicine via cuproptosis [47]. Based on the chemotherapy sensitivity analysis, it is possible that patients with high-CuRS are more responsive to platinum chemotherapy drugs. Consistently, increased drug responsiveness to olaparib and niraparib was identified in patients with high cuproptosis scores, suggesting that PARP inhibitors have potential value and providing a useful reference for combining PARP inhibitors and with cuproptosis-targeting agents to alleviate platinum resistance. Furthermore, our data show that OC patients with high cuproptosis scores had higher sensitivity to other targeted chemotherapy drugs, including VEGFR inhibitors, mTOR inhibitors, and PI3K signal inhibitors, than those with low cuproptosis scores. These results suggest that cuproptosis-related genes may predict or affect the response to chemotherapy in patients with OC.

In this study, we built a prognostic scoring model relying on cuproptosis-related DEGs in ovarian cancer samples. High CuAS samples show a more aggressive growth pattern and worse clinical outcomes than low CuRS samples. Tumor cells with different CuRS values communicate with immunocytes in a distinct manner, implying that cuproptosis activation may modulate immunocyte function. We assumed that targeting high CuRS samples may improve the patient’s prognosis, and novel potential compounds were predicted via a machine learning algorithm. The specific mechanisms underlying the function of these model genes and the correlation of prognostic and therapeutic gene signatures in ovarian cancer need further clarification. Nevertheless, our model can accurately predict the prognosis and immune status of OC patients and guide precision treatment. In summary, comprehensive bioinformatics analysis demonstrated that the CuRS model can be used to evaluate ovarian cancer aggressiveness and modulate crosstalk with immunocytes, offering a new option for chemotherapy combination treatments and response evaluation.

### Supplementary Information

Additional file1 (DOCX 4339 KB)

## Data Availability

The gene expression profiles utilized in this study are publicly accessible on TCGA (https://portal.gdc.cancer.gov/), GEO (https://www.ncbi.nlm.nih.gov/geo/), GDSC (http://www.cancerrxgene.org), CCLE (https://depmap.org/portal/download/), and RcisTarget database (https://resources.aertslab.org/cistarget/). The analytic protocols are available from the corresponding author upon reasonable request.

## Abbreviations

TCGA: The: Cancer Genome Atlas
GEO: Gene expression omnibus
CuRS: Cuproptosis-related scoring
CRGs: Cuproptosis-related genes
OC: Ovarian cancer
PARP: Poly ADP-ribose polymerase
TCA: Tricarboxylic acid
CNV: Copy number variation
KEGG: Kyoto Encyclopedia of Genes and Genomes
GO: The Gene Ontology
GSVA: Gene set variation analysis
ssGSEA: Single sample gene set enrichment analysis
ROC: Receiver operating characteristic
GDSC: Genomics of Drug Sensitivity in Cancer
CCLE: Cancer Cell Line Encyclopedia
IHC: Immunohistochemistry
TME: Tumor microenvironment
AUC: Area under the curve
