# Supplementary material for: A cuproptosis-related signature predicts prognosis and indicates cross-talk with immunocyte in ovarian cancer
Source: Discov Oncol. 2024 May 2;15:141. doi: 10.1007/s12672-024-00981-7 (PMC11065839; doi:10.1007/s12672-024-00981-7)
Supplement: Supplementary file 1 — Additional file1 (DOCX 4339 KB) [file 12672_2024_981_MOESM1_ESM.docx]

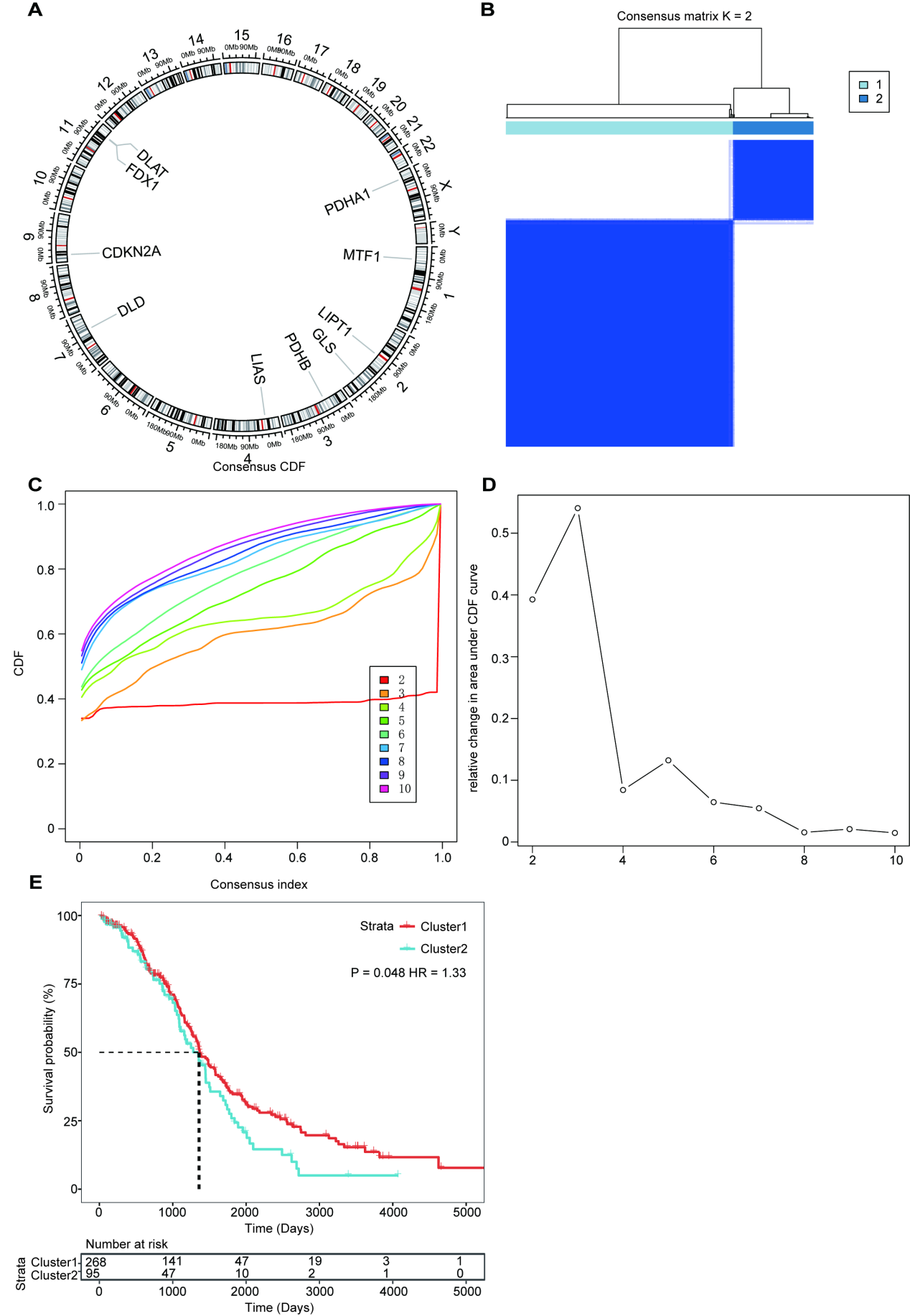


**Supplementary Figure 1** (A) The localization of CRGs CNV alterations on 23 chromosomes. (B) Consensus matrix of TCGA OC patients, k=2, using the unsupervised consensus clustering method. (C) Cumulative distribution function (CDF) when different k values. (D) The Delta area plot shows the relative change in area under the CDF curve when at a certain k value compared with k-1. (E) Kaplan-Meier analysis of the overall survival of the two clusters.


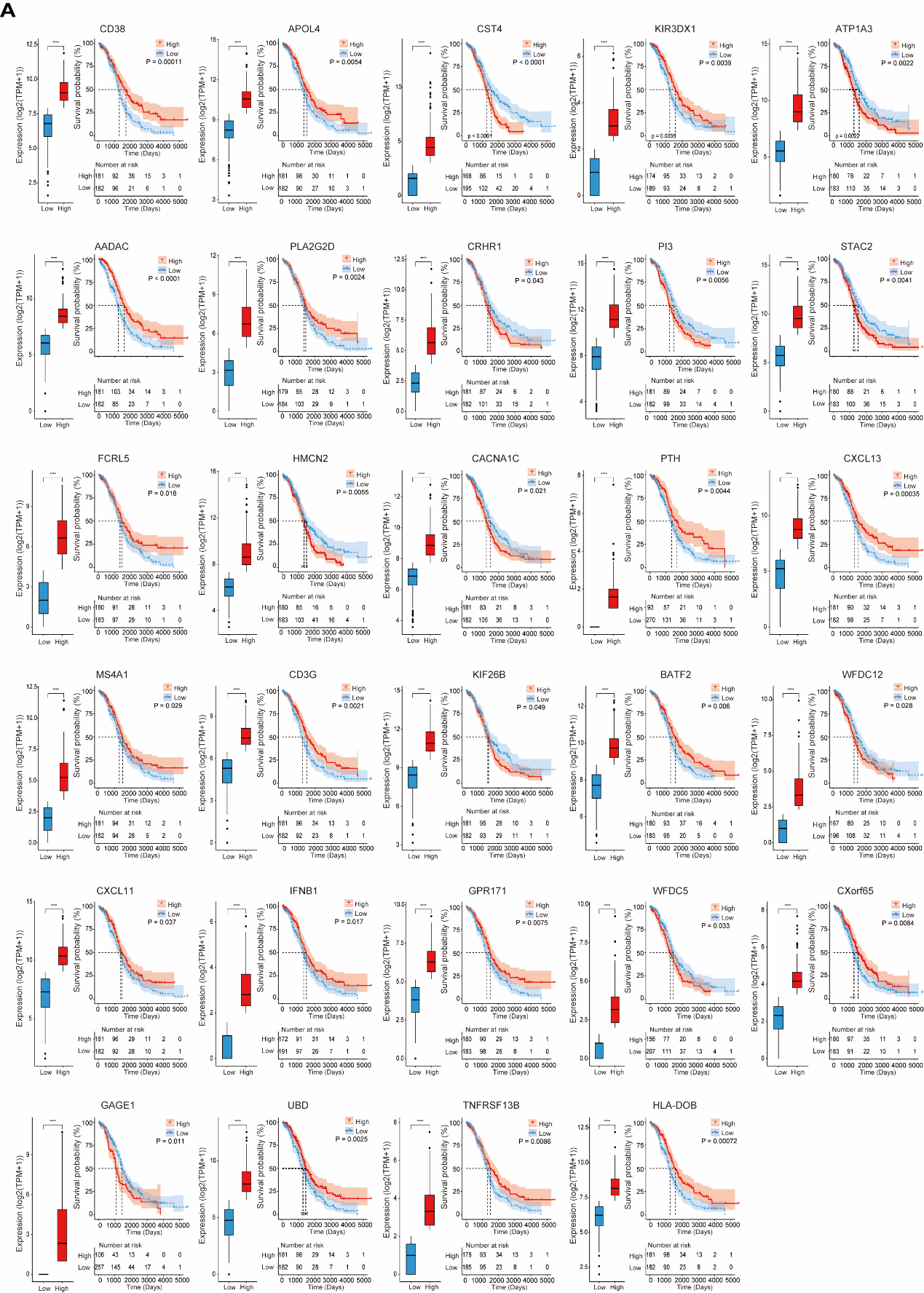


**Supplementary Figure 2** (A) Kaplan–Meier plots of differentially expressed genes in TCGA.


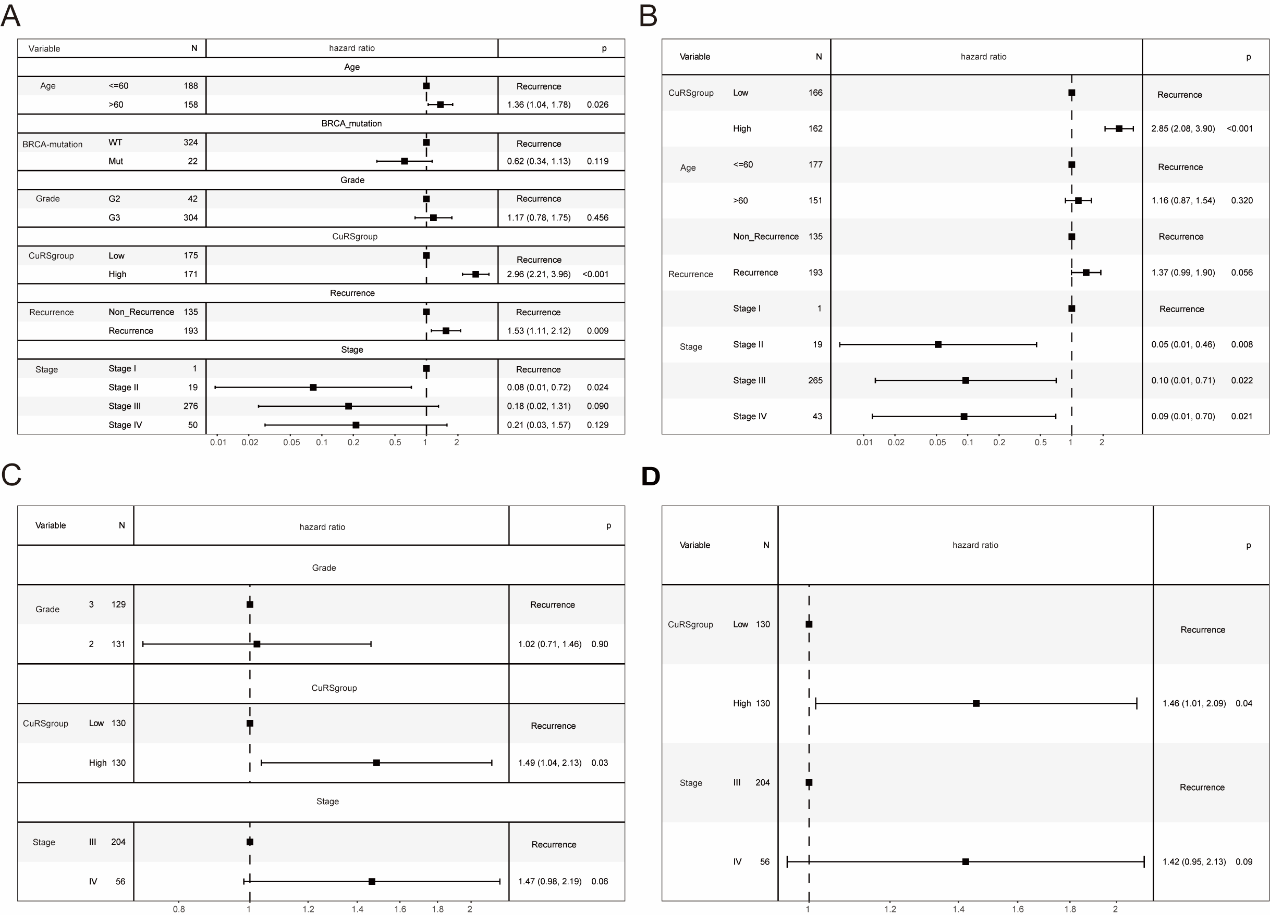


**Supplementary Figure 3** (A-D) Univariate and multivariate Cox regression analysis of the (A, B) TCGA cohort data, (C, D) GSE32062 data.

**
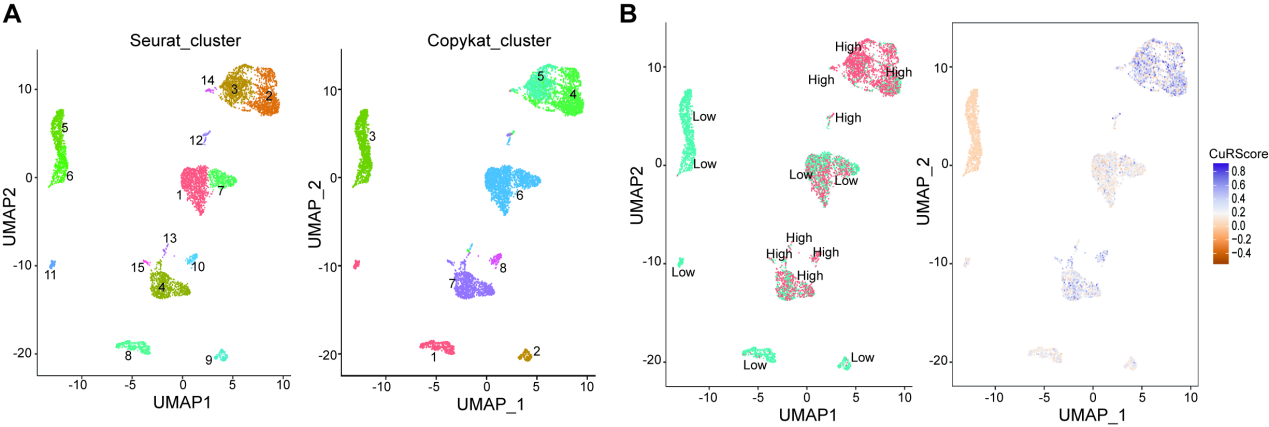
**

**Supplementary Figure 4** (A) Cluster subgroups of OC cells and subtypes based on CNV. (B) The distribution of CuRS groups in OC cells.


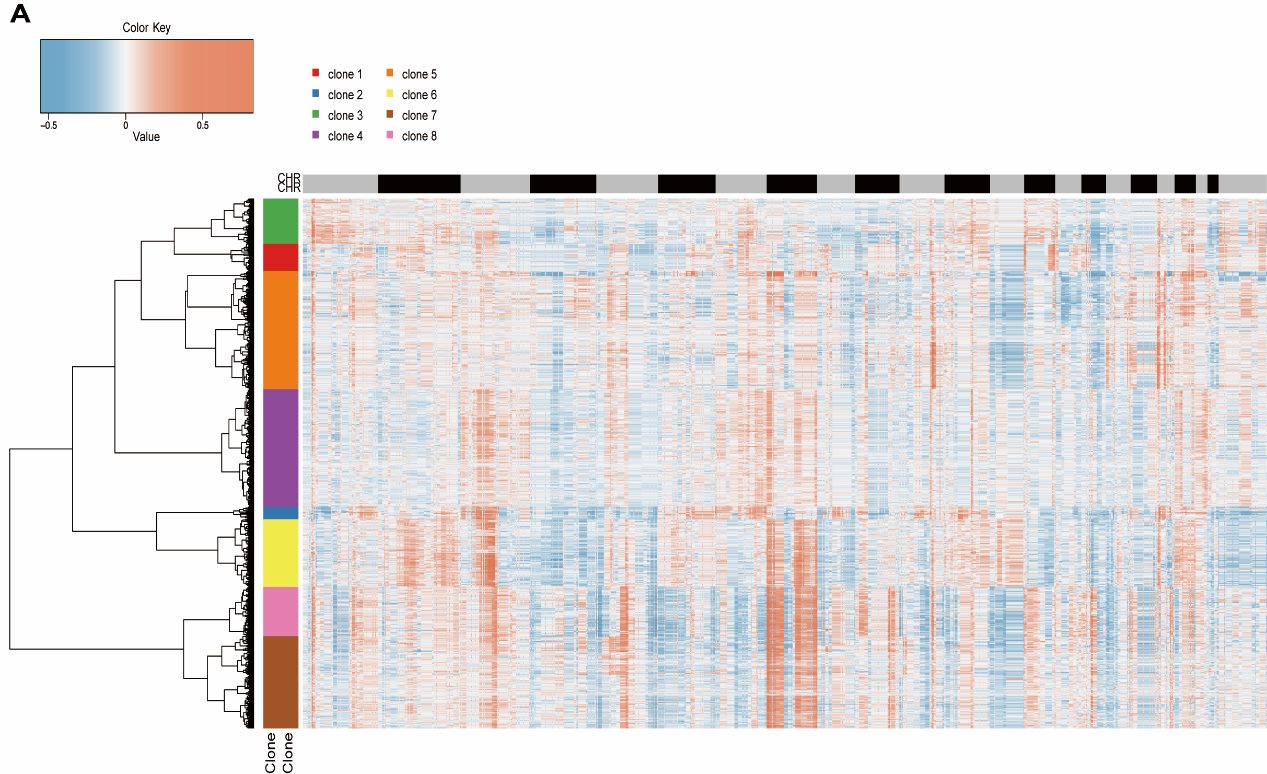


**Supplementary Figure 5 (A)** Clustering of Copy Number Variation in ovarian cancer epithelial cells.


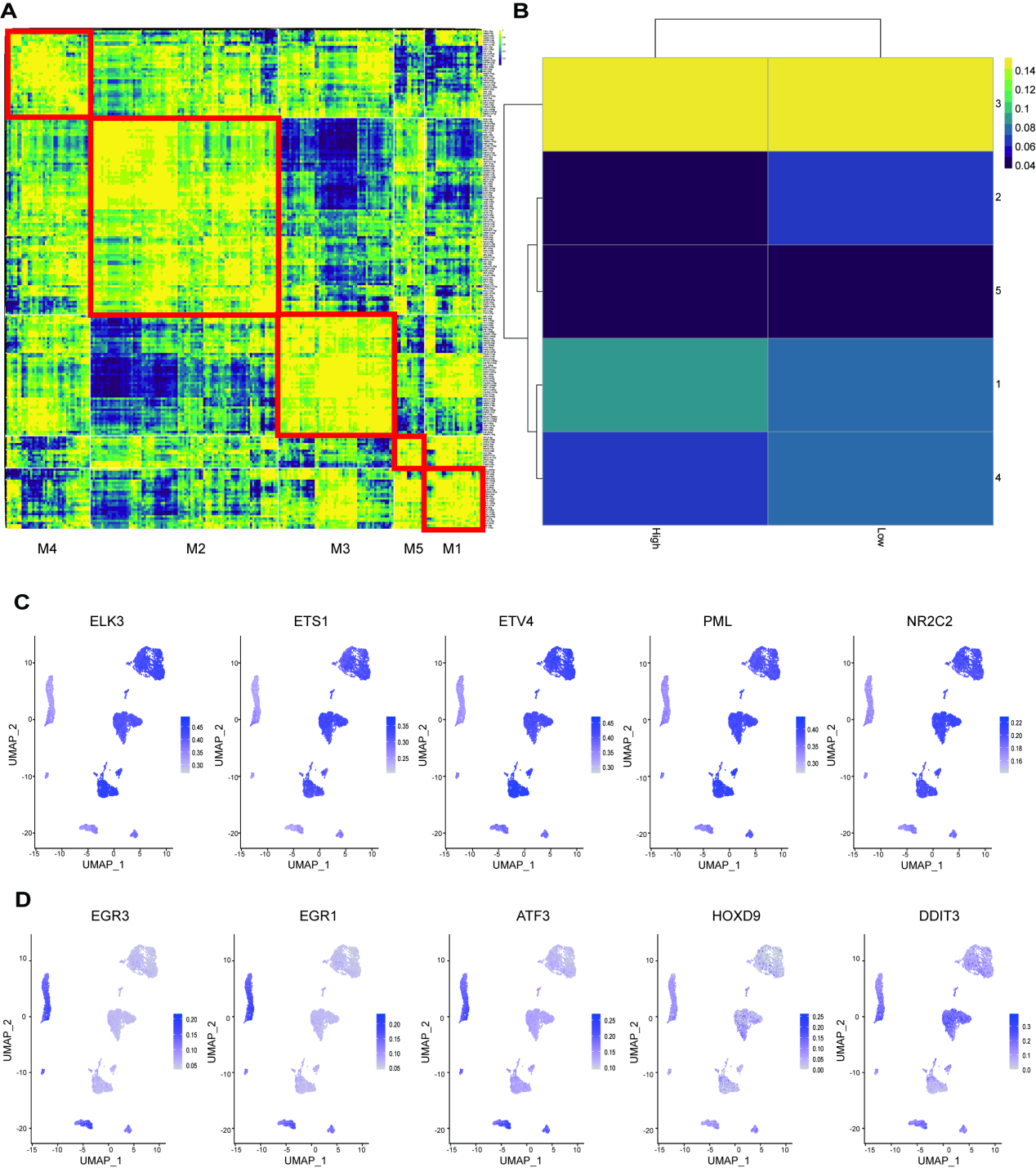


**Supplementary Figure 6** (A) According to the synergy of different transcription factors, ovarian cancer samples were divided into 5 modules. (B) The potential relationship between the CuRS scoring system and the module.(C, D) The enrichment distribution of the top 5 differentially activated TFs in the two high (C) and low (D) clusters respectively.


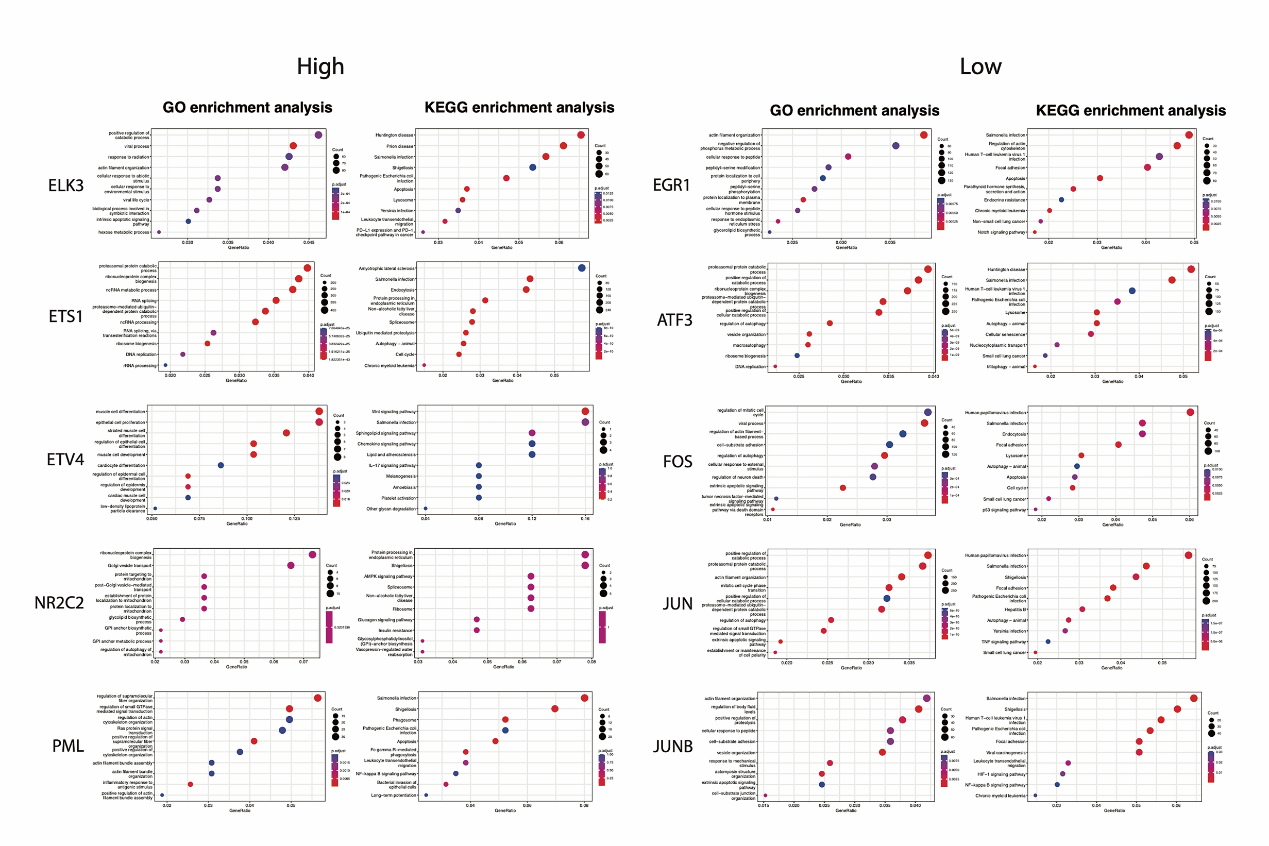


**Supplementary Figure 7** GO and KEGG enrichment analysis of the targeted genes of the top 5 differentially activated in the two high (C) and low (D) clusters respectively. TFs, transcription factors.


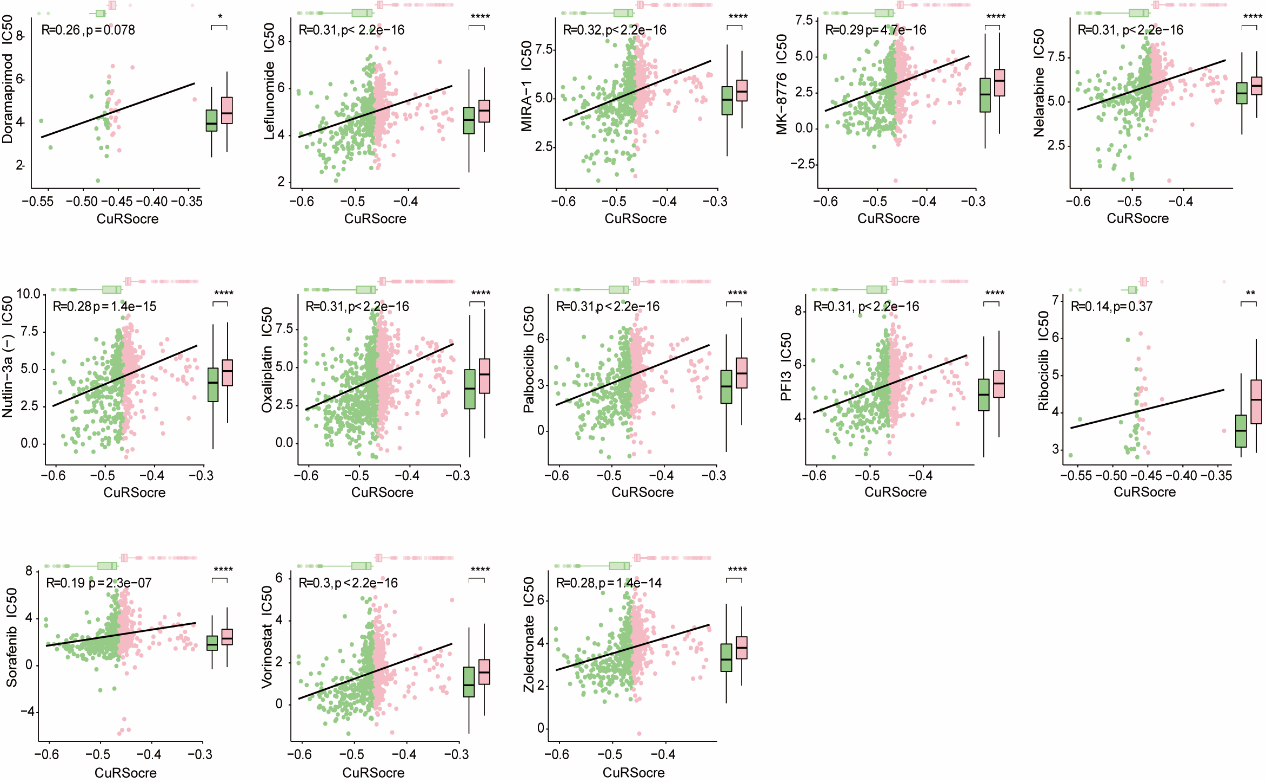


**Supplementary Figure 8** Drugs characterized by IC50 positively correlated with CuRS score in the GDSC database.
